# Preventing Healthcare-Associated Infections: Hand Disinfection Monitoring Using an Automated System in an Italian Neurological Hospital

**DOI:** 10.3390/healthcare11233018

**Published:** 2023-11-22

**Authors:** Vittorio Bolcato, Francesco Saverio Robustelli della Cuna, Giovanni Fassina, Anna Odone, Luisa Gervasio, Daniele Bosone, Lorenzo Blandi

**Affiliations:** 1 Legal Medicine Unit, I.R.C.C.S. Foundation Istituto Neurologico Nazionale C. Mondino, 27100 Pavia, Italy; vittorio.bolcato@mondino.it (V.B.); gfassina@unipv.it (G.F.); 2 Department of Drug Sciences, University of Pavia, 27100 Pavia, Italy; fsaveriorobustelli@unipv.it; 3 Legal Medicine Unit, Department of Public Health, Experimental and Forensic Sciences, University of Pavia, 27100 Pavia, Italy; 4 Public Health Unit, Department of Public Health, Experimental and Forensic Sciences, University of Pavia, 27100 Pavia, Italy; 5 Pharmacy Service, I.R.C.C.S. Foundation Istituto Neurologico Nazionale C. Mondino, 27100 Pavia, Italy; luisa.gervasio@mondino.it; 6 Health Direction, I.R.C.C.S. Foundation Istituto Neurologico Nazionale C. Mondino, 27100 Pavia, Italy; daniele.bosone@mondino.it

**Keywords:** infections, infection control, hand hygiene, preventive health services, preventive medicine, risk management, hospital administration

## Abstract

Hand hygiene plays a pivotal role in preventing Healthcare-Associated Infections (HAIs). Nevertheless, the quality of hand disinfection techniques remains suboptimal, and the reliability of assessment methodologies is notably lacking. This study aimed to evaluate hand disinfection techniques using an automated system in an Italian research hospital. Hospital employees underwent automated hand-disinfection technique assessment, according to the World Health Organization’s six-step protocol, at a basal time and two further times. Detection was carried out with a scanner that evaluated the effective hand disinfectant coverage through a fluorescent dye present in the hydro-alcoholic gel rub. The hand-hygiene technique of 222 employees was analyzed by HandInScan^®^. At the initial assessment of hand coverage with the hydro-alcoholic gel, the mean palm coverage was 82.2%, mean back coverage was 66.8%, and overall mean hand coverage was 74.5%. Then, two measurements were performed in June (t1) and December 2022 (t2). The third measurement showed an increase in hand coverage, with a mean palm coverage of 97.2%, a mean back coverage of 91.8%, and a mean hand coverage of 94.5% (*p* < 0.05). Moreover, the mean coverage of the hand-back was lower than that of the hand-palm at all times (*p* < 0.001). The automated scanner provided evidence supporting its effectiveness in enhancing hand hygiene among hospital employees. These findings have motivated researchers to conduct long-term studies, given the potential effects on HAI prevention—including their impact on HAI trends.

## 1. Introduction

The most common transmission vectors of healthcare-associated pathogens are the hands of healthcare employees (HCW); thus, hand hygiene represents the single most effective means of reducing healthcare–associated infections (HAI), with relevance for patient care safety and overall hospitalization costs [1,2,3,4].

HAIs represent the most frequent adverse event associated with patient care, contributing to significant morbidity, mortality, and financial burden in patients and healthcare systems due to direct and indirect costs—thus further representing a central indicator of health service quality [5]. Indirect costs also include those related to HAI-related litigations, a phenomenon that is growing in terms of economic impact and claim frequency [6,7,8]. Annually, the mean Italian HAI incidence rate is between 5 and 10% [9,10]. The specific incidence rate related to Neurology departments or research hospitals varies between 2.7 and 4.4% [11,12] and 3.9 to 7.4% [13], respectively. Healthcare leaders and organizations support and continuously implement hand hygiene programs within wards, focusing on HCW compliance together with long-term efficacy, but these two goals usually remain unmet [14,15,16]. Several studies have emphasized the need for the integration of HAI prevention systems in a multimodal way, highlighting the role of hand hygiene along with HCW training, personal protection equipment (PPE), and hydro-alcoholic gel use [17,18]. Other authors have studied the minimal average hand coverage for the proper hand hygiene of HCW (threshold > 85, 90, or 95%), focusing on the amount of disinfectant, the exposure time, and the disinfection technique itself, as well as the size of the hands, with different results [19,20,21]. Most of the available data on hand disinfection practice has dealt with staff compliance, assessed by various methods [22,23]. Results widely vary between 30% and 60% [24], and about 44% of physicians in neurology departments have adopted “bare below the elbows” guidelines to keep their hands disinfected [25]. If hand hygiene in its simplicity and effectiveness could prevent the spread of infections in hospitals, disinfection hand techniques remain suboptimal [26,27,28]. Additionally, technique assessment methods—based on self-observation or external observation—remain critical, with poor reliability [29,30,31].

Since the World Health Organization (WHO) recommendations on hand hygiene created in 2009, a five-dimensional model was proposed to support successful infection prevention and control (IPC): (1) Alcohol-Based Hand Rub (ABHR) at the point of care for the HCW, (2) ABHR assessment and performance feedback, (3) continuous training and education, (4) widespread reminders in the workplace, and (5) institutional safety and a supportive climate [32,33,34]. Recent studies have highlighted the value of automated systems for hand-disinfection technique assessment, albeit with limited geographic representativeness [22,35,36].

The IRCCS Fondazione Istituto Neurologico Nazionale C. Mondino (Mondino Foundation), Pavia, Italy, is an Italian research hospital dedicated to the care of patients and research in the field of neuroscience and neurological and neuropsychiatric disorders. The Mondino Foundation is equipped with an Hospital-acquired Infection Control Committee (HICC), a multidisciplinary team dedicated to the IPC function.

This study aimed to evaluate Mondino Foundation employees’ hand disinfection technique with an automated system in the context of a broad, multimodal HAI prevention project [18].

## 2. Materials and Methods

### 2.1. Hospital Context

This study was conducted at the Mondino Foundation, an Italian research hospital with 131 beds: 60 for neurological disorders, 39 for neurological rehabilitation, 22 for neuropsychiatry, and 10 technical rooms for day-hospital treatment. In 2022, there were around 3500 (2800 adult) hospitalizations and 35,000 (30,000 adult) patient days.

### 2.2. Hospital IPC Strategies

In 2022, the Mondino Foundation’s HICC collaborated with the hospital’s health direction and with the occupational health service to improve IPC activities in the hospital in a comprehensive way. The HICC planned an automated assessment of hand-disinfection technique and performance feedback, integrated with the WHO’s aforementioned five dimensional model. Disinfection protocols were revised, together with protocols on infection control and isolation, the correct use of PPE, and the management of urinary and vascular catheters. In addition, during the first half of the year, nurses were trained in urinary and vascular catheter management to prevent urinary tract and bloodstream infections. In the second half of the year, a workshop on the prevention and management of HAIs and sepsis was held. Point-of-care ABHR was provided by hydro-alcoholic gel dispensers (column or wall-mounted) located at the entrances to outpatient areas and inpatient wards, along the corridors of inpatient rooms, at the hospital entrance, in common and administrative areas, and in each outpatient room. The area supervisors were responsible for refilling the dispensers once they were empty. Posters on correct and frequent hand washing/rubbing and WHO Six Moments for hand hygiene were posted by each dispenser and in each toilet for visitors and staff [37]. The HICC, in conjunction with the hospital pharmacy service, also planned the introduction of an antimicrobial stewardship program for targeted antimicrobial therapy and submitted information regarding the overall usage of hydro-alcoholic gel (liter/1000 patient days), comparing 2022 with 2021. Concurrently, to evaluate the efficacy of the technique, the HAI rate was compared to that of the previous year.

### 2.3. Participants

In 2022, the average number of employees in the hospital was approximately 300, including 80 (26.7%) physicians (P), 90 (30%) nurses (N), 80 (26.7%) healthcare assistants (H), and 50 (16.6%) administrative employees (A). Participants were split according to their roles across the three-time intervals, as stated in Table 1. All employees participated in the study anonymously and willingly and were recognized by a numbered card. Our study was conducted as part of routine training for hospital employees in a non-clinical environment; therefore, no ethical disclosure was required. Excluding diagnostic and research facilities, measurements were conducted in inpatient wards and administrative offices.

### 2.4. Tool

**Figure 1:**
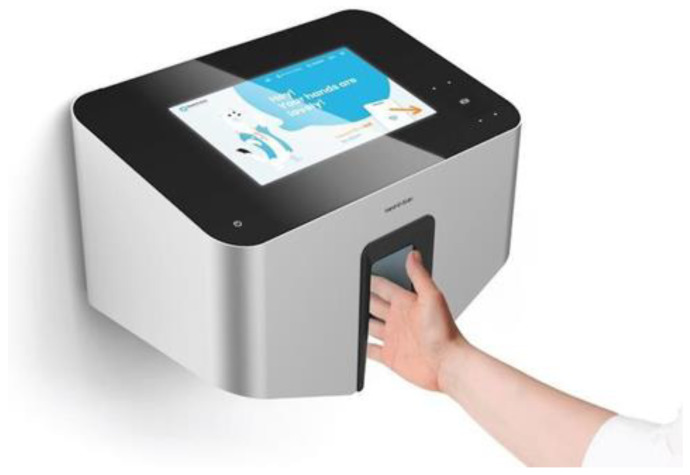
HandInScan^®^ device (Image credit: HandInScan^®^ Zrt).

The measurements were carried out by a HandInScan^®^ scanner (HandInScan Zrt., Debrecen, Hungary; Figure 1), an automated hand-hygiene technique assessment tool. The scanner tested the coverage of the disinfected hand area. The participants performed the hand rubbing technique according to the WHO six-step hand-hygiene protocol with a 2 mL (two dispensations) UV-labeled ABHR solution [38]. The solution, called Semmelweis Training Rub, contained 70% ethanol and a fluorescent dye (<0.02%) [39].

### 2.5. Statistical Analysis

A descriptive analysis was conducted for the purpose of this study. The mean, standard deviation, and absolute frequencies of quantitative variables, as well as the percentages and absolute frequencies of qualitative variables, were computed using STATA version 13 (StatsCorp, Frisco, TX, USA).

The evaluation was conducted on the coverage of the disinfected hand area; 2022 was used as the reference year, with an initial measurement in November 2021 (t0) and two additional measurements in June 2022 (t1) and December 2022 (t2).

**Figure 2:**
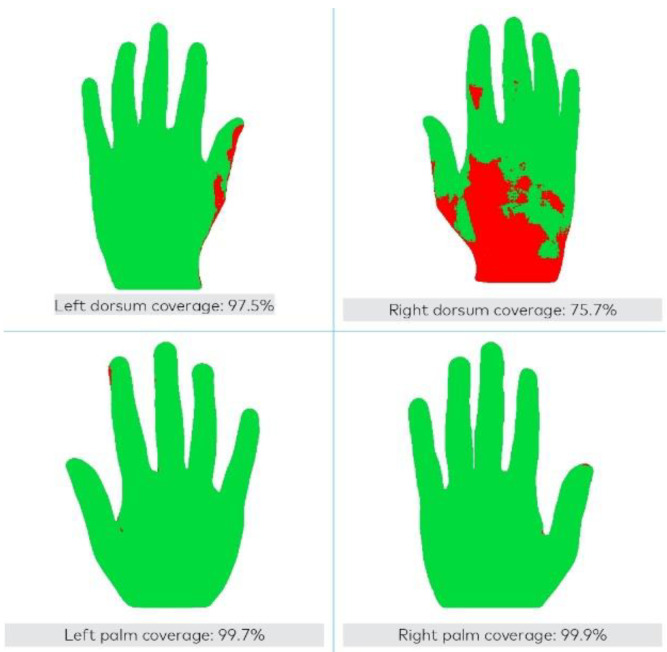
Direct visual description of the scanner device. Legend: UV-labeled ABHR solution covered in green-colored on the screen, while uncovered areas were red-colored.

The threshold for passing the test with an excellent technique was to achieve an overall disinfected hand area of 95% [20,35]. The scanner provided feedback on areas missed during disinfection. Areas adequately covered with gel appeared green-colored on the screen, while untreated areas were red-colored (Figure 2). Furthermore, the scanner displayed the overall percentage of disinfected hand area; if coverage was ≥95%, it was a pass; otherwise, the test failed. Immediate textual critical interpretation was also carried out: very dirty (0% < x < 75%), dirty (76% < x < 94%), and excellent (x ≥ 95%).

When comparing the indicators across the four groups of professionals, an analysis of variance was carried out; *p*-values less than 0.05 were considered to be significant in this study.

## 3. Results

The hand-hygiene technique of 222 employees was analyzed by the HandInScan^®^ scanner. Overall, 33% (n. 73) were in the administrative department, 27% (n. 60) were nurses, 26% (n. 56) were physicians, and 14% (n. 32) were healthcare assistants. The assessments were conducted and reported at three different time intervals (t0, t1, t2; Table 2).

At the initial (t0) assessment of hand disinfection coverage with hydro-alcoholic gel in all the participants, the mean palm coverage was 82.2% (SD = 27.4), the mean back coverage was 66.8% (30.5), and the overall mean hand coverage was 74.5% (SD = 27.6; Table 2).

The assessments at t1 (June 2022) and t2 (December 2022) showed an increase in hand coverage, together with a reduction in the standard deviation. Specifically, the mean palm coverage was 90.1% (SD = 17.0) at t1 and 97.2% (SD = 5.5) at t2 and the mean back coverage was 72.0% (SD = 27.2) at t1 and 91.8% (SD = 13.5) at t2. The mean hand coverage was 81.0% (SD = 20.7) at t1 and 94.5% (SD = 9.1) at t2.

**Figure 3:**
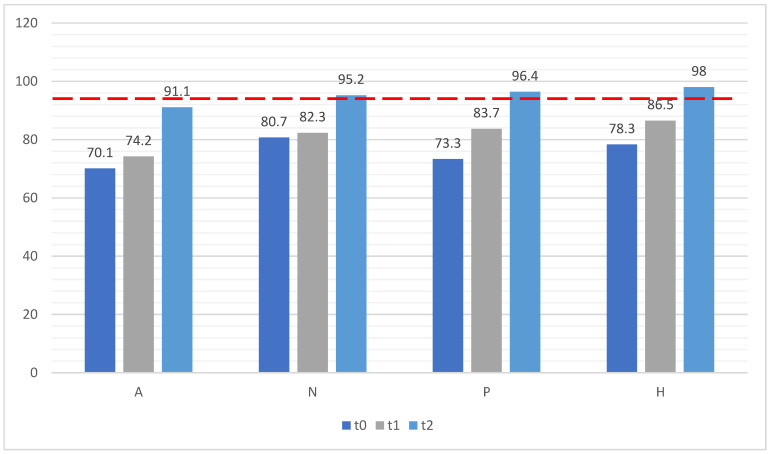
Mean overall hand coverage among hospital employees’ roles in the three assessments. Legend: red dotted line indentifies threshold target of hand coverage ≥95%.

Considering the threshold target of ≥95%, it was exceeded only at t2 for palm coverage (97.2%, SD = 5.5). Values close to the threshold (coverage ≥ 95%) were observed for the mean overall hand coverage of all employee categories at t2 (94.5%, SD = 9.1). Excluding the results for administrative employees, the values for HCW were above the threshold at t2 (96.2%, SD = 5.0; Figure 3).

Left palm coverage was higher than the right palm at t0, t1, and t2—even with a progressive gap reduction—without statistical significance. Left back coverage was lower than the right back at t0, t1, and t2—with a progressive gap reduction—without statistical significance (Table 2).

The increase in the average value for hand palm, hand back, and mean overall hand disinfection coverage for the three times showed statistical significance (*p* < 0.05). On the contrary, no statistical significance was observed between the different roles of the hospital employees (Administrative, Nurse, Physician, Healthcare assistant).

In addition, the mean coverage of the hand back was at all times lower than the hand palm, and the average increase in hand back coverage was higher than for the hand palm—both with high statistical significance (*p* < 0.001).

## 4. Discussion

In 2022, the Mondino Foundation’s HICC established a comprehensive plan for HAI prevention. The hospital administration supported the project as a strategic area for research [40,41] and development. The four aforementioned dimensions (institutional safety and a supportive climate; continuous training and education; widespread reminders in the workplace; alcohol-based hand rub at points of care for healthcare employees) were developed at the same time the HandInScan^®^ scanner was introduced.

The scanner was utilized periodically, and all employees were reminded via email one week prior to the test, to evaluate employees’ hand disinfection technique.

All participants, most notably healthcare workers, demonstrated results below the desired threshold (target ≥ 95%) as determined by the baseline measurement. Suzuki et al., in a Japanese hospital with 440 beds and 445 enrolled employees, reported a mean palm coverage of 97.8% for all hand sizes. They also reported 97.5% for the left and right hands using the WHO six-step technique, and 97.9% for both hands using the A6Sw/oI (adapted six-step without interlock) technique. The overall mean dorsal coverage varied from 93.7% to 83.0%. Coverage of more than 90% was obtained for the dorsum of both hands and both techniques performed by small hands, whereas the coverage for large hands was below 86% [19]. From the 2015 International Conference on Prevention and Infection Control, Zingg et al. reported a mean palm and dorsum coverage of 97% (95% CI, 96–98%) and 90% (95% CI, 88–93%), respectively, in 67 participants [20]. An examination of 29 university employees revealed that the palmar side had a skin coverage of 97.7% (SD 2.0%) and the dorsal side of 88.0% (SD 9.4%) [42]. Another study on 92 students resulted in 18.9% (13.8% to 24.0%) of the hand palm and 46.2% (40.0% to 52.4%) of the hand back not being covered [43].

The subsequent measurements of our study showed a gradual and progressive overall increase, reaching the desired target in specific worker categories (Figure 3). Although overall results were still insufficient at t1 and t2, this was attributable to lower performance among administrative staff who, however, did not have direct contact with patients. Rather, when only HCWs were considered during the final assessment in June 2022 (t2), results met the target threshold. Regarding the coverage of specific hand areas, the greatest increase was in the hand back, an area that is initially and typically spared by improper rubbing [36,39,44]. The mean coverage of the hand back was at all times lower than the hand palm, with high statistical significance (*p* < 0.001), and the average increase for the hand back was higher than the hand palm, with high statistical significance (*p* < 0.001). In addition, there was a statistically significant increase in the average value for the hand palm, hand back, and mean overall hand coverage in the three time intervals (*p* < 0.05).

Although there was no distinction between left-handed and right-handed individuals, and the difference was not statistically significant, the first measurement revealed a greater coverage of the left palm and right back. This suggested that right-handed individuals were more likely to pick up the gel with the left palm and rub it on the right back. The overall low initial values could be explained by the ineffectiveness of the technique compared to the WHO’s six-step protocol. Graphical and written feedback and, consequently, the improved technique, would have contributed to the increase in hand coverage as a whole.

There was no significant difference between the mean hand coverage at t0, t1, and t2 for hospital employees based on their roles, showing a similar increase over time. This suggested a non-significant difference in hand disinfection technique between healthcare and non-healthcare employees, supporting the efficacy of this hand coverage detection method with direct feedback.

Concerning the effects of training and employees’ role in the technique, the increase over the threshold target for physicians—who are typically described as frequently resistant to disinfection practices in terms of compliance and coverage [45]—was of particular interest. In a 2018 study conducted by the Department of Anesthesiology and Intensive Therapy in Hungary, the same scanner performed 604 measurements, with a median value of 99.87% coverage. The lower error rate was observed in the physiotherapy group compared to the others (physicians *p* < 0.01, nurses *p* = 0.03, assistant nurses *p* = 0.03). In the same study evaluating compliance, the lowest rate was among physicians (53.97%) [46]. Considering an overall median compliance rate of 40%, and assessing only technique compliance, the study of Erasmus et al. reported an unadjusted lower compliance rate among physicians (32%) than among nurses (48%), and lower compliance rates in intensive care units (30–40%) than in other settings (50–60%) [24]. According to Suzuki et al. [19], there was no difference based on various hospital disciplines. The improvement and the high level attained by the healthcare assistants in our study, despite not being statistically significant compared to other employees, were especially relevant as they usually have the most frequent and prolonged contact with patients. This was of particular interest in the field of neurorehabilitation (39 hospital beds in Mondino Foundation), where contact for assistance and personal hygiene is also associated with a longer inpatient stay and possible greater exposure to the hospital microbiological ecosystem [47].

Considering the overall data collected and analyzed by HICC, the 2021 hydro-alcoholic gel consumption was 317 L/1000 day, with a −10% deviation compared to the WHO minimum standard, whereas in 2022 it was 588.5 L/1000 day, with a +62% deviation compared to the WHO minimum standard. In 2022, HAIs were estimated to occur in 3.6% (3.9% only adult) of all hospitalizations, a decrease from the 5.9% (7.4% of adults) in 2021.

The high percentage of coverage, with a significant overall increase and the attainment of the target by the health professionals, along with the increase in gel consumption, could demonstrate staff commitment and support the reported decrease of HAI rates [30].

The incremental trend observed across all disciplines, including administrative ones, suggests that ongoing, integrated training plays a central role in enhancing compliance with proper hand disinfection technique; however, specific basic training is only a partial factor in technique.

The application of the scanner test training tool was simple and effective. In addition to being more stimulated by comparison with coemployees, HCWs typically recalled previous measurements and missed areas and corrected them.

## 5. Limitations

The study presented some limitations: The sample partially varied over the three measurements, as the scanner was rented for a few days per year. Thus, the staff shifts did not guarantee the participation of all the cohort members. Nonetheless, the sample was representative of the approximately 300 employees of the Mondino Foundation, owing to inter-professional diffusion and ongoing training. Moreover, considering the differences found in left-to-right and palm-to-back coverage, this could be of further interest to distinguish right-handed and left-handed hospital workers.

## 6. Conclusions

Based on the collected data, the automated and integrated assessment method for hand-disinfection technique proved to be effective in our environment. The hand-disinfection technique scanner was conducted within a multimodal IPC project, along with education/training and adequate consumption of hydro-alcoholic gel, resulting in a decrease in HAI—providing the earliest evidence of efficacy. These findings support the notion that a multi-method approach to the prevention of HAIs is preferable. Long-term efficacy must be monitored, enhancing the roles of all stakeholders—including visitors, patients, and citizens—for the safe management of the healthcare ecosystem.

## Figures and Tables

**Table 1:** Participants in automated hand-disinfection technique assessment.

	t0	t1	t2	Total
Administrative (no., %)	29 (36.25%)	20 (27%)	24 (35%)	73 (33%)
Nurse (no., %)	17 (21.25%)	28 (38%)	15 (22%)	60 (27%)
Physician (no., %)	21 (26.25%)	15 (20%)	21 (31%)	57 (26%)
Healthcare assistant (no., %)	13 (16.25%)	11 (15%)	8 (12%)	32 (14%)
Total (no.)	80	74	68	222

**Table 2:** Hand coverage assessment with hydro-alcoholic gel with the scanner.

	Left Palm Coverage (%-SD)			
	t0	t1	t2	
Administrative	80.5–32.7	89.2–17.1	97.4–4.2	
Nurse	90.1–16.1	93.6–11.6	98.2–2.9	
Physician	85.2–23.5	94.2–9.4	98.6–2.9	
Healthcare assistant	86.5–26.1	91.5–17.1	99.3–0.8	
Weighted mean	84.8–26.1	92.2–13.7	98.2–3.3	
	**Right palm coverage (%-SD)**			
	t0	t1	t2	
Administrative	76.5–34.0	84.0–22.9	93.2–13.2	
Nurse	84.5–21.8	89.1–20.0	98.0–1.9	
Physician	78.4–24.9	91.1–17.2	97.3–5.4	
Healthcare assistant	77.2 36.6	87.5–24.9	98.8–1.1	
Weighted mean	79.7–29.6	87.9–20.8	96.2–8.65	
	**Mean palm coverage (%-SD)**			
	t0	t1	t2	
Total	82.2–27.4	90.1–17.0	97.2–5.5	*p* < 0.05
	**Left back coverage (%-SD)**			
	t0	t1	t2	
Administrative	58.3–3.8	59.2–28.2	84.1–24.4	
Nurse	69.1–34.0	70.3–32.7	91.9–10.3	
Physician	65.3–34.2	75.9–29.8	93.7–9.9	
Healthcare assistant	76.3–26.7	81.4–24.5	96.0–6.5	
Weighted mean	65.3–33.0	70.1 30.2	90.2–16.8	
	**Right back coverage (%-SD)**			
	t0	t1	t2	
Administrative	65.0–31.8	64.4–21.8	89.9–16.5	
Nurse	79.3–25.5	76.1–29.0	92.6–12.3	
Physician	61.1–31.3	73.7–26.2	96.0–5.3	
Healthcare assistant	73.1–27.6	85.5–18.5	97.8–4.7	
Weighted mean	68.3–30.0	73.9–25.6	93.3–12.1	
	**Mean back coverage (%-SD)**			
	t0	t1	t2	
Total	66.8–30.5	72.0–27.2	91.8–13.5	*p* < 0.05
	**Mean overall hand coverage (%-SD)**			
	t0	t1	t2	
Administrative	70.1–31.4	74.2–19.7	91.1–13.2	
Nurse	80.7–23.2	82.3–21.7	95.2–6.0	
Physician	73.3–26.5	83.7–19.6	96.4–5.0	
Healthcare assistant	78.3–26.9	86.5–20.6	98.0–3.1	
Weighted mean	74.5–27.6	81.0–20.7	94.5–9.1	*p* < 0.05

## Data Availability

Data are contained within the article.

## References

[1] Allegranzi B., Pittet D. (2009). Role of hand hygiene in healthcare-associated infection prevention. J. Hosp. Infect..

[2] Dhar S., Sandhu A.L., Valyko A., Kaye K.S., Washer L. (2021). Strategies for Effective Infection Prevention Programs: Structures, Processes, and Funding. Infect. Dis. Clin. North Am..

[3] Umscheid C.A., Mitchell M.D., Doshi J.A., Agarwal R., Williams K., Brennan P.J. (2011). Estimating the Proportion of Healthcare-Associated Infections That Are Reasonably Preventable and the Related Mortality and Costs. Infect. Control Hosp. Epidemiol..

[4] Magill S.S., Edwards J.R., Bamberg W., Beldavs Z.G., Dumyati G., Kainer M.A., Lynfield R., Maloney M., McAllister-Hollod L., Nadle J. (2014). Multistate Point-Prevalence Survey of Health Care–Associated Infections. New Engl. J. Med..

[5] Haque M., McKimm J., Sartelli M., Dhingra S., Labricciosa F.M., Islam S., Jahan D., Nusrat T., Chowdhury T.S., Coccolini F. (2020). Strategies to prevent healthcare-associated infections: A narrative overview. Risk Manag. Healthc. Policy.

[6] Treglia M., Pallocci M., Passalacqua P., Sabatelli G., De Luca L., Zanovello C., Messineo A., Quintavalle G., Cisterna A.M., Marsella L.T. (2022). Medico-Legal Aspects of Hospital-Acquired Infections: 5-Years of Judgements of the Civil Court of Rome. Healthcare.

[7] Siracusa M., Scuri S., Grappasonni I., Petrelli F. (2019). Healthcare acquired infections: Malpractice and litigation issues. Ann. Ig. Med. Prev. Comunita.

[8] Wilcox M.H. (2004). Health-care-associated infection: Morbidity, mortality and costs. Hosp. Med..

[9] Zotti C.M., Quattrocolo F., Angelo D., Corcione S. Studio di Prevalenza Italiano sulle Infezioni Correlate all’assistenza e sull’uso di Antibiotici negli Ospedali per acuti—Protocollo ECDC. REPORT ITALIANO Point prevalence survey PPS2 2016/2017, 2018, 1–66. https://www.salute.gov.it/imgs/C_17_pubblicazioni_2791_allegato.pdf.

[10] Alrebish S.A., Yusufoglu H.S., Alotibi R.F., Abdulkhalik N.S., Ahmed N.J., Khan A.H. (2023). Epidemiology of Healthcare-Associated Infections and Adherence to the HAI Prevention Strategies. Healthcare.

[11] Arefian H., Hagel S., Heublein S., Rissner F., Scherag A., Brunkhorst F.M., Baldessarini R.J., Hartmann M. (2016). Extra length of stay and costs because of health care-associated infections at a German university hospital. Am. J. Infect. Control.

[12] Geyik M.F., Hosoglu S., Aluclu M.U., Celen M.K., Ayaz C. (2008). A 6-year prospective surveillance study for healthcare associated infections in a neurology unit. Neurosciences.

[13] Zotti C.M., Messori Ioli G., Charrier L., Arditi G., Argentero P.A., Biglino A., Farina E.C., Moiraghi Ruggenini A., Reale R., Romagnoli S. (2004). Hospital-acquired infections in Italy: A region wide prevalence study. J. Hosp. Infect..

[14] Kumar A., Keri V.C., Khan M.A., Ranja P., Rastogi N., Sahu M., Naveet W. (2021). Assessment of healthcare worker’s hand hygiene and infection prevention practices of their personal belongings in a healthcare setting: A survey in pre COVID-19 era and literature review on standard disinfection practices. J. Prev. Med. Hyg..

[15] Szilágyi L., Haidegger T., Lehotsky Á., Nagy M., Csonka E.A., Sun X., Ooi K.L., Fisher D. (2013). A large-scale assessment of hand hygiene quality and the effectiveness of the “WHO 6-steps”. BMC Infect. Dis..

[16] Lotfinejad N., Peters A., Tartari E., Fankhauser-Rodriguez C., Pires D., Pittet D. (2021). Hand hygiene in health care: 20 years of ongoing advances and perspectives. Lancet Infect. Dis..

[17] Fakih M.G., Heavens M., Ratcliffe C.J., Hendrich A. (2013). First step to reducing infection risk as a system: Evaluation of infection prevention processes for 71 hospitals. Am. J. Infect. Control.

[18] World Health Organization (2019). Minimum Requirements for Infection Prevention and Control Programmes.

[19] Suzuki Y., Morino M., Morita I., Ohiro S. (2022). Comparison of two alcohol hand rubbing techniques regarding hand surface coverage among hospital workers: A quasi-randomized controlled trial. Antimicrob. Resist. Infect. Control.

[20] Zingg W., Haidegger T., Pittet D. (2016). Hand coverage by alcohol-based handrub varies: Volume and hand size matter. Am. J. Infect. Control.

[21] Voniatis C., Bánsághi S., Ferencz A., Haidegger T. (2021). A large-scale investigation of alcohol-based handrub (ABHR) volume: Hand coverage correlations utilizing an innovative quantitative evaluation system. Antimicrob. Resist. Infect. Control.

[22] Marra A.R., Edmond M.B. (2014). New technologies to monitor healthcare worker hand hygiene. Clin. Microbiol. Infect..

[23] Zhang Y., Chen X., Lao Y., Qiu X., Liu K., Zhuang Y., Gong X., Wang P. (2023). Effects of the Implementation of Intelligent Technology for Hand Hygiene in Hospitals: Systematic Review and Meta-analysis. J. Med. Internet Res..

[24] Erasmus V., Daha T.J., Brug H., Richardus J.H., Behrendt M.D., Vos M.C., van Beeck E.F. (2010). Systematic review of studies on compliance with hand hygiene guidelines in hospital care. Infect. Control Hosp. Epidemiol..

[25] Szumska E., Czajkowski P., Zablocki M., Rozkiewicz D. (2023). A Multifaceted Approach to the “Bare below the Elbow” Concept and Hand Hygiene Compliance among Healthcare Professionals—Multicenter Population-Based Study. Int. J. Environ. Res. Public Health.

[26] Mouajou V., Adams K., DeLisle G., Quach C. (2022). Hand hygiene compliance in the prevention of hospital-acquired infections: A systematic review. J. Hosp. Infect..

[27] Ojanperä H., Ohtonen P., Kanste O., Syrjälä H. (2022). Impact of direct hand hygiene observations and feedback on hand hygiene compliance among nurses and doctors in medical and surgical wards: An eight-year observational study. J. Hosp. Infect..

[28] Pittet D. (2000). Improving Compliance With Hand Hygiene in Hospitals. Infect. Control Hosp. Epidemiol..

[29] Boyce J.M. (2021). Hand Hygiene, an Update. Infect. Dis. Clin. North Am..

[30] Pires D., Pittet D. (2017). Hand hygiene electronic monitoring: Are we there yet?. Am. J. Infect. Control.

[31] Wearn A., Bhoopatkar H., Nakatsuji M. (2015). Evaluation of the effect of hand hygiene reminder signs on the use of antimicrobial hand gel in a clinical skills center. J. Infect. Public Health.

[32] Gould D.J., Moralejo D., Drey N., Chudleigh J.H., Taljaard M. (2017). Interventions to improve hand hygiene compliance in patient care. Cochrane Database Syst. Rev..

[33] Glowicz J.B., Landon E., Sickbert-Bennett E.E., Aiello A.E., DeKay K., Hoffmann K.K., Maragakis L., Olmsted R.N., Polgreen P.M., Trexler P.A. (2023). SHEA/IDSA/APIC Practice Recommendation: Strategies to prevent healthcare-associated infections through hand hygiene: 2022 Update. Infect. Control Hosp. Epidemiol..

[34] Al-Maani A., Al Wahaibi A., Al-Zadjali N., Al-Sooti J., AlHinai M., Al Badawi A., Al Saidi A., AlZadjali N., Elshoubary W., Al-Harthi K. (2022). The impact of the hand hygiene role model project on improving healthcare workers’ compliance: A quasi-experimental observational study. J. Infect. Public Health.

[35] Németh I.A.K., Nádor C., Szilágyi L., Lehotsky Á., Haidegger T. (2022). Establishing a Learning Model for Correct Hand Hygiene Technique in a NICU. J. Clin. Med..

[36] Voniatis C., Bánsághi S., Veres D.S., Szerémy P., Jedlovszky-Hajdu A., Szijártó A., Haidegger T. (2023). Evidence-based hand hygiene: Liquid or gel handrub, does it matter?. Antimicrob. Resist. Infect. Control.

[37] WHO World Hand Hygiene Day 2022. https://www.who.int/campaigns/world-hand-hygiene-day/2022.

[38] World Health Organization Hand Hygiene Technical Reference Manual: To Be Used by Health-Care Workers, Trainers and Observers of Hand Hygiene Practices. https://apps.who.int/iris/handle/10665/44196.

[39] Lehotsky, Szilágyi L., Bánsághi S., Szerémy P., Wéber G., Haidegger T. (2017). Towards objective hand hygiene technique assessment: Validation of the ultraviolet-dye-based hand-rubbing quality assessment procedure. J. Hosp. Infect..

[40] Bolcato V., Tronconi L.P., Odone A., Blandi L. (2023). Healthcare-acquired SARS-CoV-2 infection: A viable legal category?. Int. J. Risk Saf. Med..

[41] Bolcato V., Tronconi L. (2023). Pietro Le infezioni correlate all’assistenza: Stato dell’arte. Sanità Pubblica Priv..

[42] Wang C., Jiang W., Yang K., Sarsenbayeva Z., Tag B., Dingler T., Goncalves J., Kostakos V. (2022). A System for Computational Assessment of Hand Hygiene Techniques. J. Med. Syst..

[43] Hautemanière A., Diguio N., Daval M.C., Hunter P.R., Hartemann P. (2009). Short-term assessment of training of medical students in the use of alcohol-based hand rub using fluorescent-labeled hand rub and skin hydration measurements. Am. J. Infect. Control.

[44] Reilly J.S., Price L., Lang S., Robertson C., Cheater F., Skinner K., Chow A. (2016). A Pragmatic Randomized Controlled Trial of 6-Step vs 3-Step Hand Hygiene Technique in Acute Hospital Care in the United Kingdom. Infect. Control Hosp. Epidemiol..

[45] Lamping J., Tomsic I., Stolz M., Krauth C., Chaberny I.F., von Lengerke T. (2022). Do task and item difficulty affect overestimation of one’s hand hygiene compliance? A cross-sectional survey of physicians and nurses in surgical clinics of six hospitals in Germany. Antimicrob. Resist. Infect. Control.

[46] Mogyoródi B., Szabó M., Dunai E., Mester B., Hermann C., Gál J., Iványi Z. (2019). Implementation of immediate feedback system into hand hygiene practice in the intensive care unit. Orv. Hetil..

[47] Baccolini V., D’Egidio V., De Soccio P., Migliara G., Massimi A., Alessandri F., Tellan G., Marzuillo C., De Vito C., Ranieri M.V. (2019). Effectiveness over time of a multimodal intervention to improve compliance with standard hygiene precautions in an intensive care unit of a large teaching hospital. Antimicrob. Resist. Infect. Control.

